# Macular dystrophies: clinical and imaging features, molecular genetics and therapeutic options

**DOI:** 10.1136/bjophthalmol-2019-315086

**Published:** 2019-11-08

**Authors:** Najiha Rahman, Michalis Georgiou, Kamron N Khan, Michel Michaelides

**Affiliations:** 1 Moorfields Eye Hospital, London, UK; 2 Institute of Ophthalmology, UCL, London, UK; 3 Ophthalmology Department, St James’s University Hospital, Leeds, UK

**Keywords:** macular dystrophy, retina, Stargardt disease, ABCA4, STGD, Best disease, BEST1, X-linked retinoschisis, XLRS, RS1, autosomal dominant drusen, ADD, EFEMP1, Sorsby fundus dystrophy, TIMP3, pattern dystrophy, PRPH2, gene therapy, pharmacological therapy, stem cells

## Abstract

Macular dystrophies (MDs) consist of a heterogeneous group of disorders that are characterised by bilateral symmetrical central visual loss. Advances in genetic testing over the last decade have led to improved knowledge of the underlying molecular basis. The developments in high-resolution multimodal retinal imaging have also transformed our ability to make accurate and more timely diagnoses and more sensitive quantitative assessment of disease progression, and allowed the design of optimised clinical trial endpoints for novel therapeutic interventions. The aim of this review was to provide an update on MDs, including Stargardt disease, Best disease, X-linked r etinoschisis, pattern dystrophy, Sorsby fundus dystrophy and autosomal dominant drusen. It highlights the range of innovations in retinal imaging, genotype–phenotype and structure–function associations, animal models of disease and the multiple treatment strategies that are currently in clinical trial or planned in the near future, which are anticipated to lead to significant changes in the management of patients with MDs.

## Introduction

Macular dystrophies (MDs) are a group of inherited retinal disorders that cause significant visual loss, most often as a result of progressive macular atrophy. They are characterised by bilateral, relatively symmetrical macular abnormalities that significantly impair central visual function.^1^ While the fundus findings may be predominantly located at the central retina, in the vast majority of MDs there is psychophysical, electrophysiological or histopathological evidence of more widespread, generalised retinal involvement. Over the last decade, there have been multiple advances that now provide us a better understanding of the genetic mechanisms and associated pathophysiology underlying each subtype of MD. This has thereby facilitated the development of therapeutic strategies to slow/halt progressive visual loss or potentially restore a degree of visual function.^2^

This review provides an update on monogenic MD and discusses the the most common subtypes, including Stargardt disease (STGD), Best disease (BD), X-linked retinoschisis (XLRS), autosomal dominant drusen (ADD), Sorsby fundus dystrophy (SFD) and pattern dystrophy (PD). For each subtype, detailed clinical features, retinal imaging, molecular genetics, and ongoing or planned clinical trials, including gene therapy, cellular therapy and pharmacological treatments, are discussed. In the online supplementary table, we summarise the genetics and the novel interventions in trial for the presented diseases. Developmental macular disorders are not included in this review but have been described in detail previously.^3^

10.1136/bjophthalmol-2019-315086.supp1 Supplementary data

### Stargardt disease

**Figure 1:**
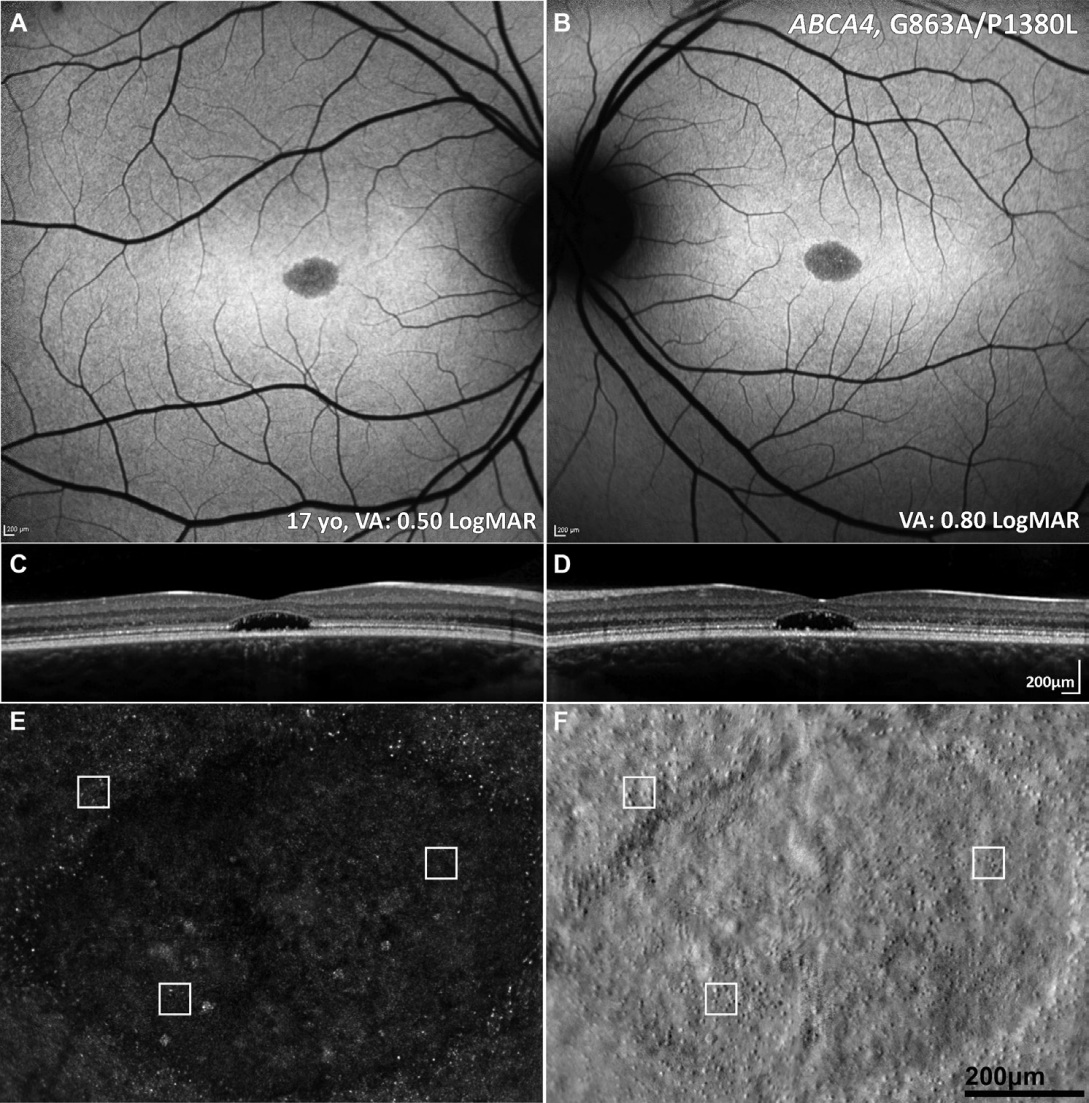
Multimodal imaging of a 16-year-old adolescent with molecularly confirmed STGD. (A, B) Fundus autofluorescence images showing a central area of decreased signal at the macula. (C, D) Corresponding horizontal transfoveal optical coherence tomography scans showing central loss of the ellipsoid zone. (A–D) Findings are symmetrical between the eyes. (E, F) Adaptive optics scanning light ophthalmoscopy of the right eye. Confocal detection (A) and split detection (B) over the foveal lesion in exact coregistration. The white box of 55×55 µm denotes regions of interest in the exact same locations in the two images. Cones are more reliably identified using split detection (B) due to the poor waveguiding of the outer segments in confocal imaging (A). VA, STGD, Stargardt disease; VA, visual acuity.

STGD is the most common MD, affecting 1:8000 to 1:10 000 people worldwide.^4^ It is characterised by the widespread deposition of lipofuscin (bisretinoids) in the retinal pigment epithelium (RPE), which gives rise to the classical fundus appearance of retinal flecks. The spectrum of disease is highly variable, in terms of the age of onset, clinical features, rate of progression and extent of retinal involvement, ranging from isolated macular disease (figure 1A, B) to generalised cone and rod system involvement.^5–10^

#### Clinical features

The classical presenting phenotype of STGD is of retinal flecks, predominantly located around the macula, with variable midperipheral distribution, most clearly visualised with fundus autofluorescence (FAF) imaging. Over time, macular atrophy develops, causing increasing visual impairment with disease progression. Patients typically present with reduced central visual function, with highly variable visual acuity (VA), depending on the degree of foveal involvement.^8^ Colour vision abnormalities, photophobia and slow dark adaptation are also common clinical presentations. The severity of visual impairment is also dependent on the age of disease onset; that is, early-onset STGD is associated with more severely compromised vision and poorer outcomes.^7 9 11^ Onset is most common in childhood, with the next peak being early adulthood, and least frequently in later adulthood (late-onset/foveal-sparing STGD).^5–8 12^ There is slow progressive loss of retinal structure and function over time; however, there is marked variability both within and between families, suggesting that other important factors influence phenotype, including genetic modifiers and the environment.^6–8^

The electrophysiological phenotype is variable and has been categorised into three groups based on the full-field electroretinography (ffERG) with all patients having an abnormal pattern electroretinography (ERG) indicative of macular dysfunction.^13^ Group 1 is characterised by normal ffERG, in keeping with isolated macular dysfunction; patients in group 2 have abnormal cone ERGs and normal rod ERGs, indicative of generalised cone system dysfunction; and patients in group 3 have both abnormal cone and rod ERGs, indicating generalised cone and rod system dysfunction. These ERG groups have been shown to have prognostic value in STGD, with group 1 being associated with a more favourable prognosis (and an 80% likelihood of not developing generalised retinal dysfunction over a 10-year period) than groups 2 and 3, with group 3 associated with the worst prognosis, with inexorable progression resulting in worse VA and additional peripheral visual loss.^14 15^ In addition to ERG, function has been shown to be impaired with scotopic microperimetry.^16^

#### Retinal imaging

A characteristic pattern of areas of increased and decreased signals on FAF imaging is seen in STGD. Fundus fluorescein angiography (FFA) demonstrates a dark choroidal phase due to masking from lipofuscin deposition in the RPE but has been superseded by FAF and optical coherence tomography (OCT). Autofluorescence imaging may serve as a monitoring tool, and decreased autofluorescence area measurements can be used as a structural outcome for interventional clinical trials that aim to slow disease progression.^17^ Ultrawide field FAF has now allowed for the classification of the posterior pole and peripheral fundus FAF findings in STGD.^18–21^ OCT is an invaluable modality in all macular diseases, which in STGD identifies and sensitively quantifies the degree and extent of outer retinal loss (photoreceptor layers) and RPE atrophy (figure 1C, D). Moreover, it can identify childhood-onset STGD before symptoms are noted by demonstrating hyper-reflectivity at the base of the foveal outer nuclear layer.^5^ It also demonstrates excellent visualisation of the anatomical level of the retinal flecks that may correlate with visual function.^8 9 22–26^ Flecks are not present in the fovea, suggesting an alternative mechanism for central cone death; cones recycle vitamin A via Muller cells, and in in early stages, yellow (intraretinal) dots are observed. The earliest signs of atrophy appear to spare the foveola and are juxtafoveal, even in childhood-onset cases (where this very early stage has been seen).^5^

The application of multimodal imaging, using FAF, OCT and optical coherence tomography–angiography (OCTA), has advanced our understanding of pathogenesis and elucidated the outer retinal cellular sequential loss in STGD, with profound implications for therapeutic targets, treatment strategies, and both clinical trial design and endpoints.^27 28^ For example, by employing en face OCT and OCTA, and detailed assessment of progression using both FAF and OCT in large well-characterised cohorts, it has been shown that photoreceptor loss is likely to precede RPE degeneration, both of which contribute to choriocapillaris loss.^5 6 29^ Cellular imaging in vivo using adaptive optics (AO) has identified increased photoreceptor spacing and reduced cone densities^30 31^; moreover, it has shown that cones can be accurately visualised, counted and tracked over time (figure 1E, F).^32^

#### Genetics

While *ABCA4-*associated STGD (STGD1, OMIM #248200) is an autosomal recessive condition, there are two autosomal dominant MDs that have phenotypical features that can overlap with some of the presentations of STGD1 (including bull's-eye maculopathy). The first is due to disease-causing variants in *ELOVL4* (STGD3, OMIM #600110), and the second is more common and due to variants in *PROM1* (STGD4, OMIM #603786).

ABCA4 is part of the ATP-binding cassette family that is involved in the active transport of various substrates across cellular membranes. The pathophysiology of STGD is a result of defective ABCA4 transport of retinoids (as part of the visual cycle), resulting in an abnormal accumulation of lipofuscin and related toxic by-products (including A2E) in the RPE and photoreceptors, with subsequent cell dysfunction and death overtime.^33^ The *abca4^−/^* ^−^ knockout mouse model has been critical in elucidating this sequence of events and has also served as a model to test therapeutic approaches (see further).^34^

The degree of ABCA4 inactivation relates to the ABCA4 disease spectrum, with STGD associated with milder sequence variants and thereby often milder inactivation of ABCA4 and a milder phenotype, compared with severe variants, resulting in he complete absence of ABCA4 function and thereby more severe disease, such as cone and rod dystrophy. The vast allelic heterogeneity (more than 1000 disease-causing ABCA4 variants) makes genotype–phenotype correlations challenging.^35^ However, there is increasing evidence that onset relates to the severity of the underlying *ABCA4* variants, with childhood-onset STGD being associated with more deleterious variants (including nonsense variants) compared with adult-onset or the later onset foveal-sparing STGD (more frequently missense variants).

#### Management and avenues of intervention

Patients are offered low-vision aids/assistive technologies to help optimise their vision, provided with adequate social support and advised on healthy living/diet, including not to take vitamin A supplements and to reduce UV exposure to potentially slow progression.

Pharmacotherapy directly or indirectly targeting the visual cycle has been developed, including the complement-mediated response to accumulated by-products of the visual cycle.^36^ Drugs such as soraprazan, emixustat, ALK-001, STG-001, fenretinide and A1120 are visual cycle modulators that impede formation of A2E and lipofuscin either by slowing the rate of vitamin A dimerisation (ALK-001) or by competitive inhibitory mechanisms on the retinal binding protein-4 (STG-001, fenretinide and A1120), or by modulating the activity of RPE65 (emixustat). Many of these drugs are in phase I/II or III trials (emixustat: NCT03772665 and NCT03033108, ALK-001: NCT02402660). Avacincaptad pegol, a complement C5 inhibitor, is also being investigated in a phase II trial (NCT03364153), as is antioxidant supplementation (saffron) (NCT01278277).

Preclinical studies in gene replacement that showed phenotypical improvement in *abca4^−/^* ^−^ mice have encouraged the development of human gene therapy clinical trials,^37 38^ with ongoing trials employing a lentiviral vector (NCT01736592 and NCT01367444).^39^ Adeno-associated virus (AAV) has many advantages over lentiviral vectors but has limited cargo capacity; several strategies are being explored to try and accommodate the large *ABCA4* gene and thereby commence AAV-based gene therapy trials.^37 39^

In advanced disease, cell replacement strategies offer potential benefit. The only phase I/II clinical trial (NCT01469832) of human embryonic stem cell (hESC)-derived RPE cells in STGD has been completed.^40 41^ Findings from the UK site of this trial identified subretinal hyperpigmentation consistent with the survival of viable transplanted hESC-derived RPE cells. Borderline improvements in VA were noted in 4 of 12 patients; however, microperimetry did not demonstrate evidence of functional benefit at 12 months. Further trials are anticipated, including evaluation of combined RPE and photoreceptor transplants, which are derived from either hESCs or induced pluripotent stem cells (iPSCs).

### Best disease

BD is the second most common MD, affecting approximately 1 in 10 000.^4^ BD is an autosomal dominant condition associated with disease-causing variants in *BEST1*.^42^ *BEST1* sequence variants also account for at least four other phenotypes, including adult vitelliform MD,^43^ autosomal dominant vitreochoroidopathy,^44^ autosomal recessive bestrophinopathy (ARB)^45^ and retinitis pigmentosa.^46^

#### Clinical features

**Figure 2:**
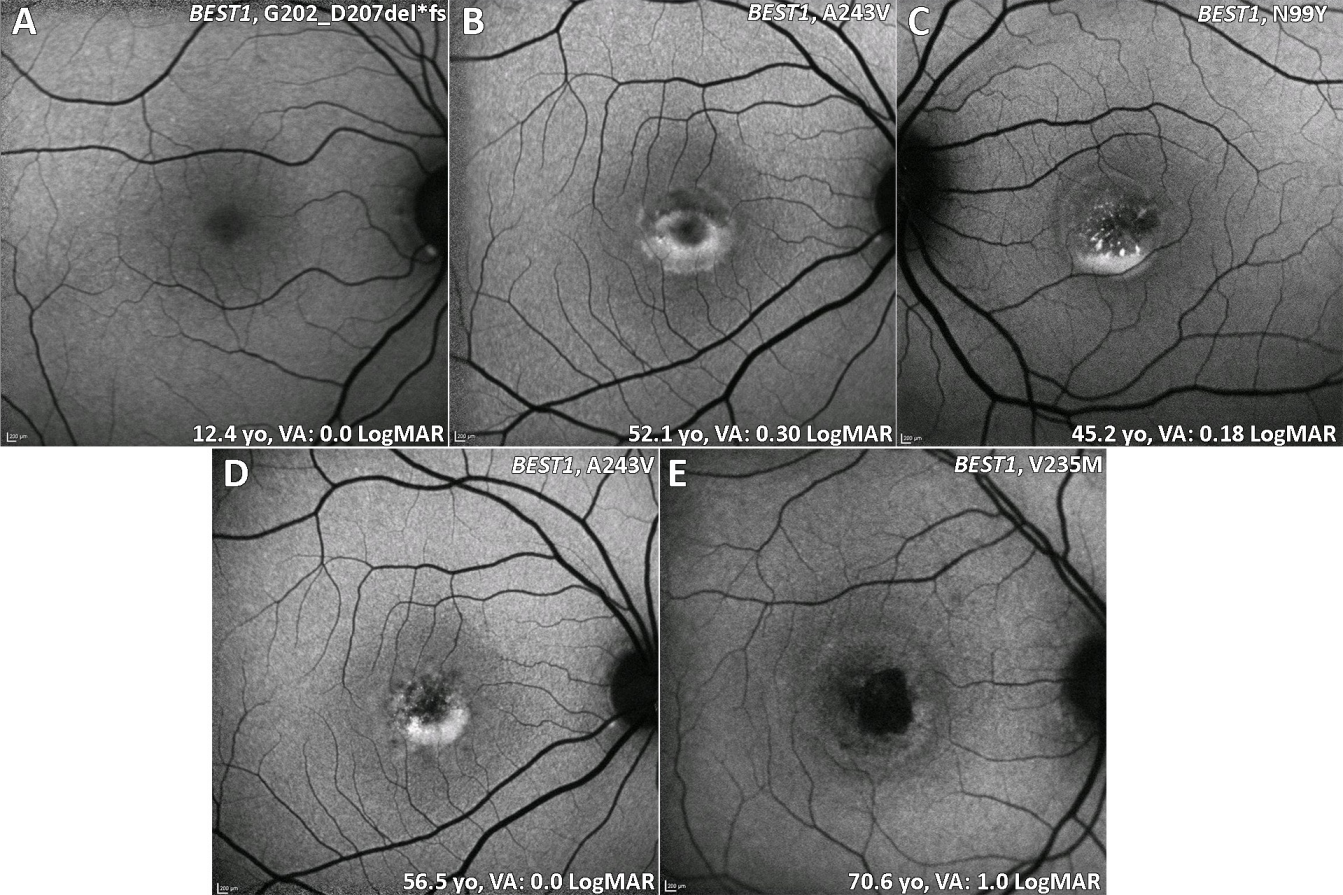
BD (BEST1) fundus autofluorescence imaging of four patients at different stages. (A) Stage 1: normal previtelliform presentation. (B) Stage 2: vitelliform lesion, classical appearance of a single, symmetrical egg yolk-like lesion at the fovea. (C) Stage 3: pseudohypopyon, material gravitates inferiorly within the vitelliform lesion. (D) Stage 4: vitelliruptive stage, the material ‘scrambles’. (B and D) Images are from the same patient over 4.4 years of follow-up. (E) Stage 5: macular atrophy. BD, Best disease; VA, visual acuity.

The onset of BD is generally in early childhood up to late teenage years.^47^ It is important to note that BD is often associated with hypermetropia, which needs correction in childhood to reduce the likelihood of amblyopia, with ARB typically associated with a greater degree of hypermetropia and a high risk of angle-closure glaucoma, thereby often necessitating prophylactic intervention to prevent acute angle closure. The classical appearance of BD is the single, bilateral symmetrical egg yolk-like (vitelliform) lesion at the fovea (stage 2, figure 2B). Stage 1 is characterised by a normal fundus or minimal RPE changes (previtelliform) (figure 2A). Over time, this lesion can start to undergo resorption, progressing to a 'pseudohypopyon' appearance, with the subretinal yellow material gravitating inferiorly within the lesion (stage 3, figure 2C). Stages 1 and 2 are associated with normal VA, and patients can be identified coincidentally or during a family survey, with VA reduction starting from stage 3 onwards. Further progression can result in a 'vitelliruptive stage' due to further breakdown of subretinal material (stage 4, figure 2D). End-stage disease (stage 5) is characterised by either atrophy (figure 2E),^48^ sub-RPE fibrosis or choroidal neovascularisation (CNV). Even though fundus features can be classified into different stages, there is rarely a predictable progression from one to the other, with the prognosis often being relatively good in many patients, despite a marked intrafamilial variability.^4^ Uniocular cases of BD have been also described.^49^

Diagnosis can be most reliably confirmed with molecular genetic testing. The electro-oculogram (EOG) can also be helpful, demonstrating a light peak to dark trough ratio of less than 1.5. Patients with BD have normal full-field ERGs, unlike in ARB. However, rarely, the EOG may be normal in both BD and ARB, further highlighting the importance of genetic testing.^50^

#### Retinal imaging

OCT best identifies the subretinal vitelliform lesion, which is associated with a high signal on FAF imaging (figure 2).^51^ Subretinal fluid (SRF), which waxes and wanes over time, is also common. The increased signal on FAF can help to distinguish inherited causes of vitelliform lesion compared with non-inherited acquired disorders. The fibrosis that can occur in advanced BD has been described as resembling a ‘circus tent’ due to its exaggerated height and unusual height to base ratio.^52^ OCTA has suggested this fibrosis to have a neovascular origin.^53^ Fibrotic lesions appear hypoautofluorescent on FAF. OCTA is particularly useful in identifying CNV in vitelliform disorders, including BD, where FFA can be very challenging to interpret.

#### Genetics

Disease-causing missense variants in *BEST1* (OMIM #607854) most commonly underlie BD. Most of the variants identified occur in the first half of the gene. Haploinsufficiency of those variants is tolerated and is not associated with the BD phenotype. Specific variants are reported to cause less severe phenotype than others (eg, Ala243Val).^54^

*BEST1* encodes bestrophin-1, a protein localised to the basolateral membrane of RPE cells. One of the critical functions of bestrophin-1, a calcium-sensitive chloride channel, is to regulate the ionic environment in the RPE and/or subretinal space. Dysregulation of this function, in part due to an alteration in the adhesiveness of the interphotoreceptor matrix to the RPE, results in the vitelliform deposition seen in BD.^55–58^ Variants in other genes can also result in a similar vitelliform phenotype as seen in BD, including *PRPH2*, *IMPG1* 59 60 and *IMPG2*.^59^

In ARB, compound heterozygous null variants in *BEST1* are observed. Unlike dominant diseases, ARB is characterised by *multifocal* vitelliform deposits, often associated with SRF and/or cystoid macular oedema.

#### Progression and management

Prognosis can often be relatively good in BD, although associated with marked intrafamilial and interfamilial variabilities. However, progressive resorption of subretinal material can be associated with slow central visual deterioration, unless BD is complicated by CNV, which can result in acute marked visual loss. Acute visual loss/metamorphopsia, retinal haemorrhage and *intraretinal* fluid should raise suspicion of CNV and investigation; SRF is unhelpful, given it is often observed in BD not complicated by CNV (thereby, SRF is also not a useful indicator of CNV treatment response). Intravitreal bevacizumab has been found to be very effective, with improvement in structural and functional measurements, in direct contrast to observation alone.^61^ Unlike other causes of CNV, those associated with inherited retinal disease often require limited injections; usually one or two are sufficient.

#### Avenues of intervention

Canine models of ARB have been successfully rescued with AAV-mediated gene replacement.^62 63^ Research avenues for *BEST1*-dominant disease are at present limited.

### X-linked retinoschisis

XLRS is the most common form of juvenile-onset retinal degeneration in male adolescents. Female carriers are almost always unaffected, with only a single case report of a symptomatic girl.^64^

#### Clinical features

**Figure 3:**
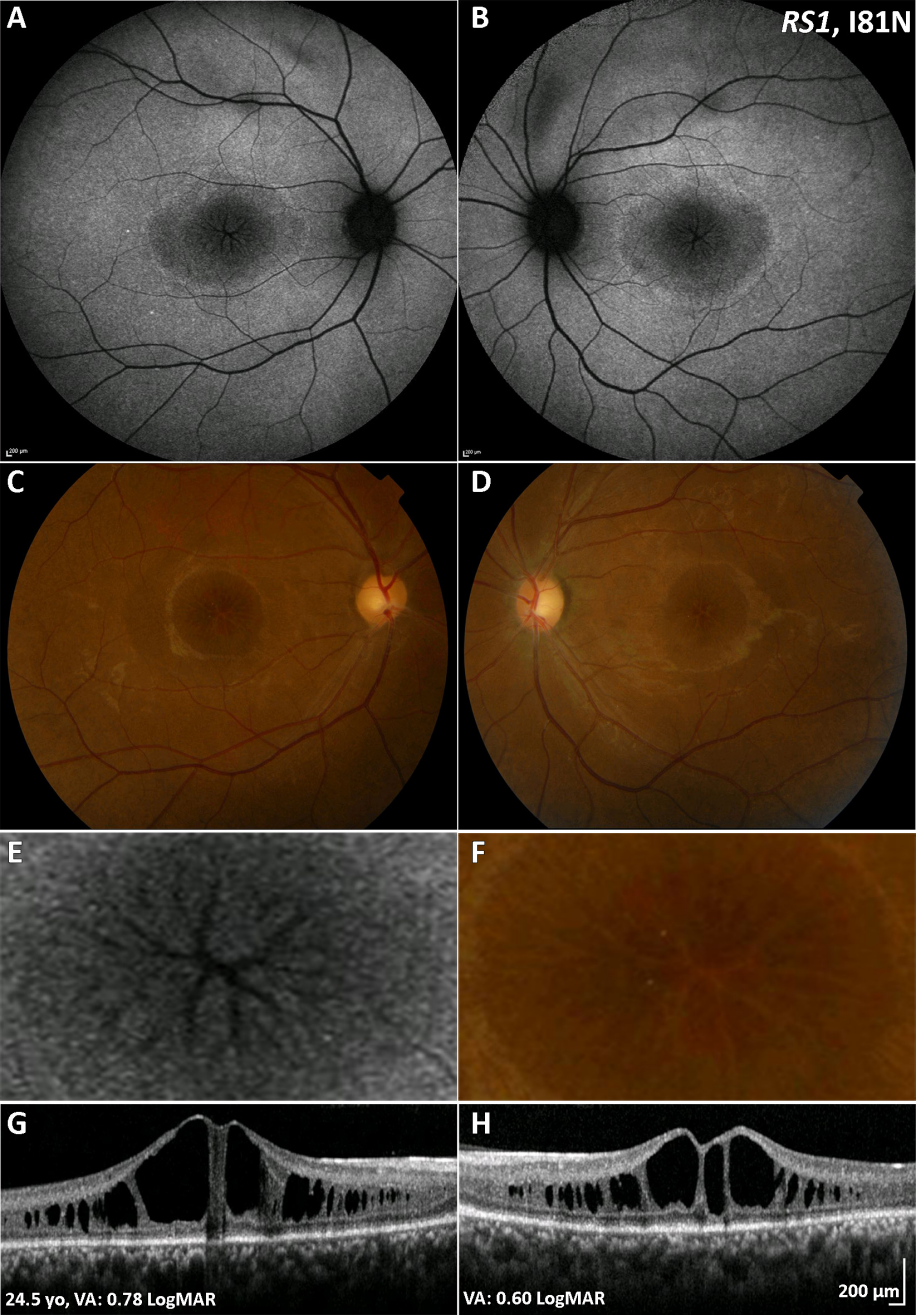
XLRS (*RS1*) multimodal imaging of a 25-year-old patient with XLRS. (A, B) Fundus autofluorescence imaging, with a central decreased signal over the macula and a rim of increased signal, absent signal in a ‘spoke wheel’ pattern over the central fovea in both eyes, shown in greater magnification in (E) for the right eye. (C, D) Colour fundus photographs with the same pattern as (A, B). (F) Greater magnification of the foveal centre of the right eye. (G, H) Transfoveal optical coherence tomography scans showing the extent of schisis cavities in the outer retina. VA, visual acuity; XLRS, X-linked retinoschisis.

XLRS typically presents in the first to second decade in a variety of ways, including with poor VA, strabismus, anisometropia and 'unexplained visual loss'. Prognosis is variable but can be relatively good in childhood if not complicated by retinal detachment (RD) or vitreous haemorrhage (VH), which are both associated with a poor prognosis in childhood or adulthood.^4 65^ Older adults may experience slow VA loss due to the development of macular atrophy. ‘Spoke-wheel’ folds of the macula (macular schisis) are the hallmark feature of XLRS (figure 3A–D). Approximately 50% of male adolescents also have peripheral retinal changes, including schisis, metallic sheen, pigmentary disturbance, white spiculations, vitreous veils and neovascularisation. Patients with peripheral retinoschisis have an increased risk of VH and RD.^66^ Bullous XLRS can be congenital or may develop soon after birth, with strabismus being the most common presenting feature. Such cases can be complicated later in life by RD, which may be tractional or a Coats-like exudative detachment.^67^

The ERG in XLRS typically shows a reduced b:a-wave ratio during dark-adapted bright flash recording, also known as an ‘electronegative ERG’. There appears to be a strong correlation between anatomical (OCT) and functional (ERG) measures between eyes in XLRS; often both can be relatively stable over time, in keeping with a relatively stationary natural history in many patients.^68^

#### Retinal imaging

Macular schisis can be easily missed on clinical examination, making multimodal imaging invaluable. OCT can readily identify splitting of the inner and outer retinal layers (figure 3E, F), and FAF imaging shows a spoke-wheel appearance of concentric areas of high-signal and low- signal intensity. Rarely macular OCT can be normal/near normal, and peripheral changes are then the only clue to clinical diagnosis. Vascular abnormalities such as vascular sheathing and neovascularisation have been described in XLRS, and ultrawide field imaging with FFA and OCTA may thereby be helpful.^69^ AO imaging has identified increased and irregular cone spacing within the foveal schisis; however, the presence of preserved waveguiding cones at the fovea and macular regions may indicate a good potential for successful rescue with intervention.^27 70^

#### Genetics

Disease-causing variants in *RS1* (OMIM #312700) underlie XLRS, with the encoded cell-surface protein, retinoschisin-1, expressed in photoreceptors and bipolar cells, having a role in retinal cell adhesion. *RS1* variants disrupt the subunit assembly of the protein and lead to alteration of normal retinal cell adhesion, thus resulting in splitting of the neural layers of the retina. Molecular screening of *RS1* is needed to identify female carriers, given their lack of retinal phenotype, which is unusual in X-linked retinal disorders.

#### Progression and management

Carbonic anhydrase inhibitors (CAIs) have been shown to be useful in managing schisis in XLRS. The first study that reported the use of CAIs in XLRS treated eight patients with topical CAI (2% dorzolamide) and observed a reduction in foveal thickness in seven of the eight patients, with five also having VA improvement ≥7 letters.^71^ Similar results were seen in 66% of patients in a cohort of 36 treated with either topical or oral CAIs.^72^ There has also been a disconnect reported between VA improvement and lack of structural change.^73^ It has been proposed that CAIs may alter the fluid-transport mechanism across the RPE, resulting in a reduction of fluid contained within the macular schisis.^74^

#### Avenues of intervention

Intravitreal *RS1* gene replacement in knockout mice has resulted in functional ERG improvement.^75 76^ This has led to two phase I/II XLRS gene therapy trials (NCT02416622 and NCT02317887) delivering gene replacement intravitreally. The former trial has ceased due to marked ocular inflammation associated with intravitreal delivery, while the latter has added additional agents to the standard oral steroids used in subretinal gene supplementation trials to address the uveitis adverse events.

### Pattern dystrophy

**Figure 4:**
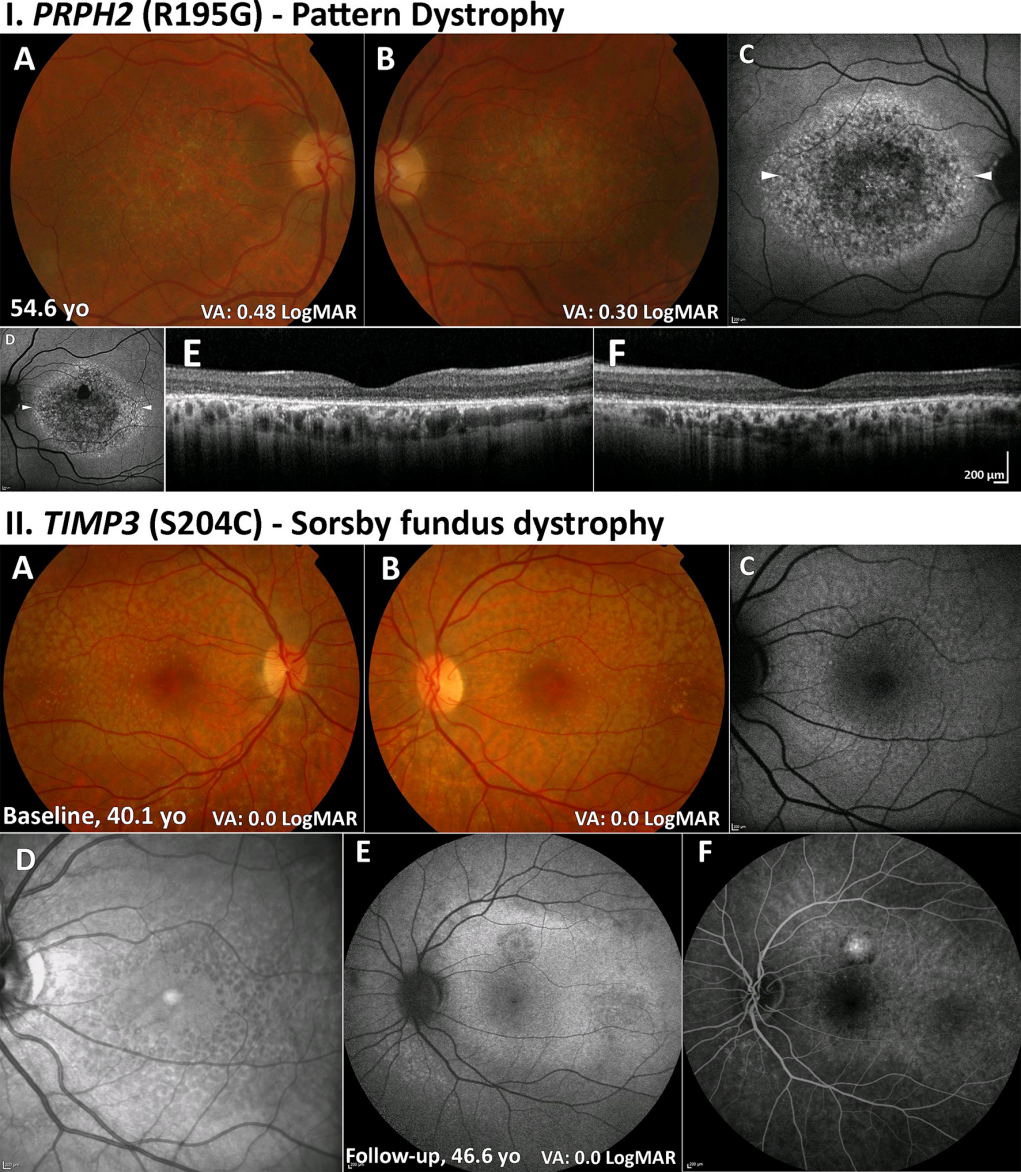
PD (*PRPH2*) and SFD (*TIMP3*). (I) PD (A, B): colour fundus photographs with bull’s-eye maculopathy-like retinal pigment epithelial changes and fine mottled symmetrical depigmentation of the macula. (C, D) FAF imaging displays a florid speckled appearance with areas of increased and decreased macular autofluorescence. The white arrowheads denote the location of the optical coherence tomography line scans shown in (E) and (F). (E, F) Extensive disruption of the ellipsoid zone and retinal pigment epithelium hypertrophy in both eyes. II. SFD (A, B): colour fundus photographs with fine symmetrical drusen-like deposits at the posterior pole. (C) FAF imaging of the left eye displays patchy ill-defined increased autofluorescence. (D) Infrared image over the same location as (C) readily depicting the drusen-like deposits. (E) FAF image after 6.5 years of follow-up showing a superior area of CNV. (F) Fundus fluorescein angiography showing an inactive CNV. CNV, choroidal neovascularisation; FAF, fundus autofluorescence; PD, pattern dystrophy; SFD, Sorsby fundus dystrophy; VA, visual acuity.

PD describes a group of disorders characterised by variable distributions of pigment deposition at the level of the RPE (figure 4I A, B).

#### Clinical features

Patients with PD often present in the fourth to fifth decades, either following routine optometry review or having noticed a mild limitation in the central vision. PD is usually associated with a good prognosis, unless rarely complicated by CNV (highly responsive to anti-VEGF agents). Several different fundus appearances have been described based on the variable patterns of deposition, including butterfly-shaped pigment dystrophy and reticular dystrophy. ERGs in PD are generally normal or only mildly subnormal.

#### Retinal imaging

The deposits in PD are typically hyperautofluorescent on FAF (figure 4I C, D) and may result in a characteristic speckled pattern.^77^ As these changes are at the level of RPE, subretinal hyper-reflective material is seen on OCT (figure 4I E, F).

#### Genetics

PD is an autosomal dominant condition most often due to variants in *PRPH2* (OMIM #169150). Other genes such as *BEST1* 78 and *CTNNA1* 79 have also been associated with PD phenotypes. *PRPH2* encodes peripherin-2, a multimeric structural protein that establishes and maintains the morphology of photoreceptor outer segment (OS) discs.^80^ Abnormal peripherin-2 found in patients with PD results in an ultrastructural alteration of OS discs with an abnormal whorl-like arrangement histopathologically.^81^

#### Progression and management

Given that PD typically has a later age of onset, it can be misdiagnosed as age-related macular degeneration (AMD). Retinal imaging, including FAF, OCT and OCTA, is helpful to distinguish between PD and AMD in order to ensure the appropriate management is followed. PD has increased parafoveal superficial and deep vessel densities on OCTA, hyper-reflective material in the subretinal space on OCT and hyperautofluorescence on FAF imaging.^82 83^

#### Avenues of intervention

Successful integration and material transfer of donor-derived or stem cell-derived cone photoreceptors in Prph2^rd2/rd2^ murine models of the disease are promising.^84^

### Sorsby fundus dystrophy

SFD is a rare ADD-associated MD often leading to bilateral central visual loss in the fifth decade of life.

#### Clinical features

Early signs of SFD are the macular yellowish-grey drusen-like deposits at the level of the Bruch membrane, which preferentially accumulate along the temporal arcades (figure 4II A–D). Patients may be asymptomatic at this stage; however, difficulty with dark adaptation can be an early symptom. The deposits progress over time to include the central macula. Visual loss can be secondary to slow atrophic degeneration at the macula, which can also extend peripherally. CNV is a common complication, often resulting in severe VA loss (figure 4II C–F).

#### Retinal imaging

FAF imaging may identify a broad ill-defined increase in signal in the peripheral macula in early disease, with subretinal drusenoid deposits, reticular pseudodrusen, that spare the central fovea, clearly depicted on infrared imaging (figure 4II). OCT may identify drusen-like deposits and delineate Bruch membrane thickening and is valuable in the diagnosis of CNV. OCTA has also been shown to capture early CNV changes without the need for FFA.^85^

#### Genetics

SFD is associated with missense variants in the tissue inhibitor of metalloproteinase-3 (*TIMP3*) gene (OMIM #188826). These substitutions usually create a new cysteine residue, with p.Ser204Cys being the the most common. TIMP3 is an inhibitor of matrix metalloproteinases, which play an important role in the regulation of extracellular matrix (ECM) turnover. Mutant TIMP3 accumulates within Bruch membrane, disrupting the homeostasis of ECM remodelling and interfering with the normal critical functions of Bruch membrane, choroid and RPE.^86^

#### Progression and management

Prognosis in SFD is generally poor due to development of atrophy and/or CNV (figure 4II E, F). Prompt use of anti-VEGF injections may improve outcome for SFD complicated by CNV.^87–90^

#### Avenues of intervention

Early attempts at treating SFD involved oral vitamin A at 50 000 IU/day, with a short‐term reversal of night blindness in patients at early stages of disease.^91^ Due to the potential toxicity of long-term high-dose vitamin A and reports of lack of efficacy at lower doses (15 000 IU/day) in advanced disease, vitamin A is not a widely used treatment.^92^ Currently, no animal or cell culture model capable of recapitulating human SFD is available.^93^ Patient-derived iPSC–RPE models can provide a suitable platform for investigating SFD.^94^

### Autosomal dominant drusen

ADD is an autosomal dominant condition characterised by drusen-like deposits at the macula, which may be in a radiating or honeycomb-like appearance.

#### Clinical features

**Figure 5:**
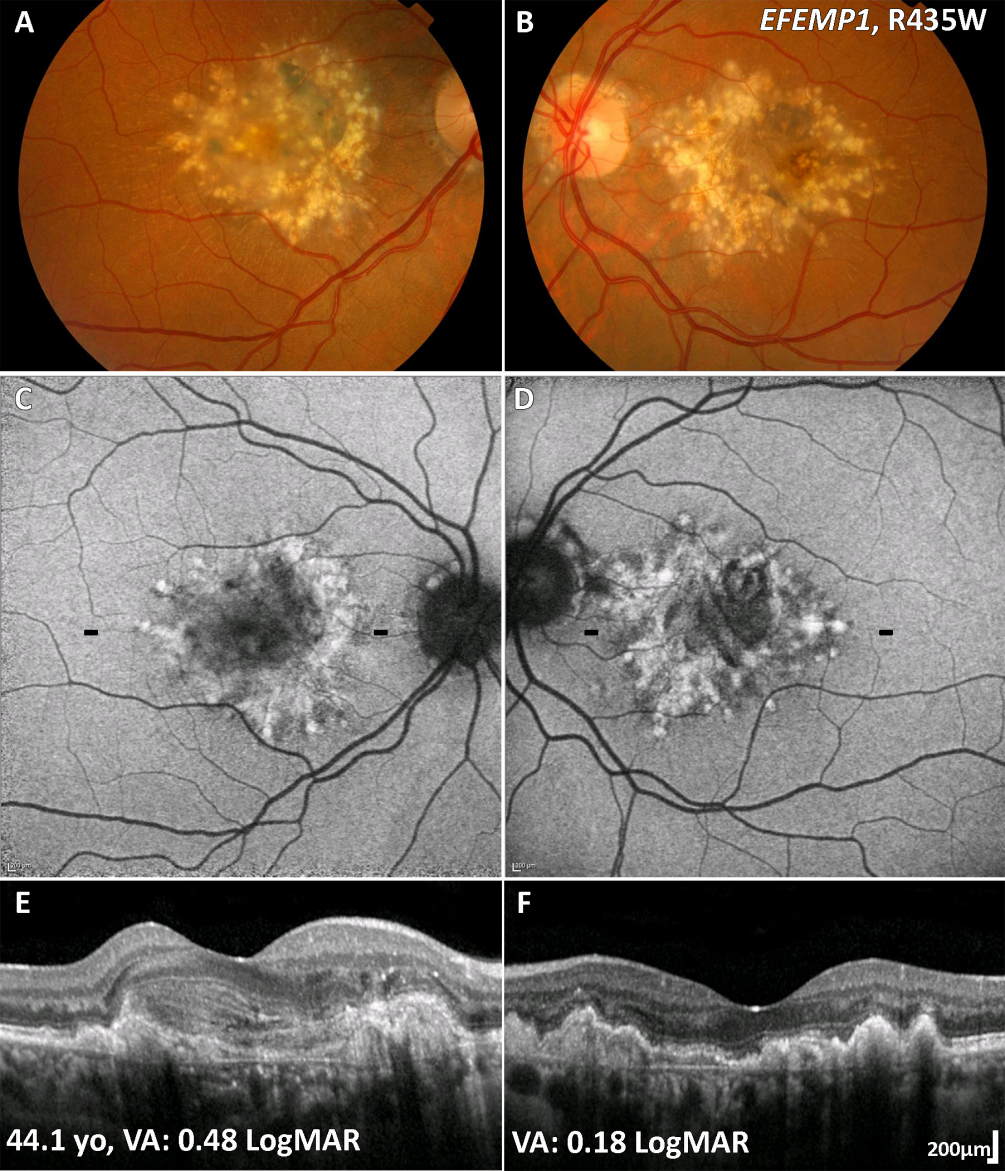
ADD (*EFEMP1*) multimodal imaging of a 44-year-old patient. (A, B) Colour fundus photographs with characteristic radial distribution of macular drusen. Note the CNV in the right eye (A). (C, D) Fundus autofluorescence images with the drusen associated with an increased signal. The black dashes denote the location of the OCT line scans shown in (E) and (F). (E, F) In both eyes hyper-reflective thickening of the retinal pigment epithelium-Bruch membrane complex, with disrupted photoreceptor integrity. (E) CNV is seen associated with a reduction in VA. ADD, autosomal dominant drusen; CNV, choroidal neovascularisation; *EFEMP1*, EGF-containing fibulin-like extracellular matrix protein-1; VA, visual acuity.

ADD encompasses both 'Doyne honeycomb retinal dystrophy' and 'Malattia Leventinese', with the latter associated with a characteristic radial distribution of macular drusen (figure 5A, B). The drusen in ADD typically abut the optic nerve head.^95^ Visual loss may occur in ADD due to the development of a variable degree of central atrophy or, rarely, can be complicated by CNV (figure 5A, C and E).^95^ There is marked interfamilial and intrafamilial variabilities observed in terms of retinal appearance, severity and progression.^95^

#### Retinal imaging

In contrast to drusen in AMD, the drusen-like deposits in ADD are hyperautofluorescent on FAF (figure 5C, D).^95 96^ On OCT, drusen-like deposits are seen as a hyper-reflective thickening of the RPE–Bruch membrane complex,^97^ with disrupted photoreceptor integrity (figure 5F).^98^ OCTA can be valuable to diagnose CNV in ADD.^99^

#### Genetics

A single missense variant, p.Arg345Trp, in EGF-containing fibulin-like extracellular matrix protein-1 (*EFEMP1*) (OMIM #601548) is responsible for ADD. EFEMP1 is a member of the fibulin family that encodes for fibulin-3 (F3). The p.Arg345Trp substitution in F3 results in a sub-RPE membranous accumulation of debris associated with signs of complement activation and RPE atrophy in a mouse model of ADD.^100^

#### Progression and management

Progression is highly variable and some patients maintain useful reading vision until later in life. Progression is usually slow and secondary to macular atrophy. When rarely complicated by CNV, anti-VEGF agents are highly effective.

#### Avenues of intervention

Similar to SFD, no animal or cell culture model capable of recapitulating human ADD is available, with patient-derived iPSC–RPE models also being explored for investigating ADD.^94^ Efemp1 knockout mice do not develop sub-RPE deposits following exposure to environmental stressors (high-fat diet/laser or high-fat diet/cigarette smoke), which may suggest that deletion of Efemp1 may have a protective role in ADD.^101^

## Conclusions

Our understanding of MD has significantly evolved over the last decade, resulting in improved diagnosis (both more accurate and at earlier stages of disease), better advice on prognosis and therapeutic opportunities. These advancements have been based on the availability of novel high-resolution multimodal imaging and better molecular genetic testing. While multiple therapeutic avenues are being explored in autosomal recessive STGD and XL recessive XLRS (summarised in online supplementary table), far less progress has been made in the autosomal dominant disorders BD and PD, which together account for a significant burden of disease. This is most likely due to the greater challenges associated with developing genetic therapies for AD disease, compared with the gene replacement approach required in AR/XL disorders. This unmet need is likely to be addressed in the next decade with the rapid evolution of gene silencing/editing technology.
