# Supplementary material for: Macular dystrophies: clinical and imaging features, molecular genetics and therapeutic options
Source: Br J Ophthalmol. 2019 Nov 8;104(4):451–60. doi: 10.1136/bjophthalmol-2019-315086 (PMC7147237; doi:10.1136/bjophthalmol-2019-315086)
Supplement: Supplementary data [file bjophthalmol-2019-315086supp001.pdf]

Supplementary Table: Genetics of Retinal Dystrophies and Avenues of Intervention (as of September 2019)

Disease	OMIM #	Gene	Inheritance Mode	Avenues of Human Intervention		
				Drug	Mechanism of Action	Clinical Trial
**Stargardt disease**						
STGD1	248200	*ABCA4*	AR	**Visual cycle modulators**		
				A1120	Competitive inhibitory mechanisms on the retinal binding protein-4	
				Fenretinide	Competitive inhibitory mechanisms on the retinal binding protein-4	
				STG-001	Competitive inhibitory mechanisms on the retinal binding protein-4	
				Soraprazan	Removal of lipofuscin in RPE cells	Phase II
				Emixustat	Modulating the activity of RPE65	Phase III (NCT03772665) and Phase IIa (NCT03033108)
				ALK-001	Decrease the rate of Vitamin A dimerization	Phase II (NCT02402660)
				**Antioxidant**		
				Saffron	Antioxidant Supplement	Phase I/II (NCT01278277)
				**C5 inhibitor**		
				Avacincaptad pegol	Complement C5 inhibitor	Phase II (NCT03364153)
				**Gene Therapy**		
				SAR 422459	Lentiviral vector gene supplementation	Phase I/IIa (NCT01367444) and Phase I/II (NCT01736592)
				**Cell Replacement**		
				MA09-hRPE	Subretinal human embryonic stem cell (hESC) derived RPE cells	Phase I/II (NCT01469832)
STGD3	600110	*ELOVL4*	AD		NA	
STGD4	603786	*PROM1*	AD		NA	
**Best disease**	607854	*BEST1*	AD		NA	
**Retinoschicis**	312700	*RS1*	X-linked	**Carbonic anhydrase inhibitors**	Alter the fluid transport mechanism across the RPE	
				**Gene Therapy**	Intravitreal AAV gene supplementation	Phase I/II (NCT02416622 and NCT02317887)
**Pattern dystrophy**	169150	*PRPH2*	AD		NA	
**Sorsby fundus dystrophy**	188826	*TIMP3*	AD		NA (Anti-VEGF for comlicated cases with CNV)	
**Autosomal dominant drusen**	601548	*EFEMP1*	AD		NA (Anti-VEGF for comlicated cases with CNV)	

AR: autosomal recessive, AD: autosomal dominant, NA: not available, RPE: retinal pigment epithelium, VEGF: vascular endothelial growth factor, CNV: choroidal neovascularization
